# Bilateral Well Leg Compartment Syndrome Localized in the Anterior and Lateral Compartments following Urologic Surgery in Lithotomy Position

**DOI:** 10.1155/2018/2328014

**Published:** 2018-11-14

**Authors:** Tatsuya Yamamoto, Atsuhiro Fujie, Hidenori Tanikawa, Atsushi Funayama, Kentaro Fukuda

**Affiliations:** Department of Orthopedic Surgery, Saiseikai Yokohamashi Tobu Hospital, 3-6-1 Shimosueyoshi, Tsurumi Ward, Yokohama, Kanagawa 230-0012, Japan

## Abstract

Well leg compartment syndrome (WLCS) is a rare but severe complication after the surgery in lithotomy position. We present a case of bilateral WLCS that occurred after the prolonged urologic surgery in lithotomy position. A 50-year-old man complained of severe bilateral lower leg pain and swelling sixteen hours after the surgery. Physical examination, elevated serum creatine kinase value, contrasting computed tomography, and elevated compartment pressure strongly suggested the development of bilateral WLCS localized in the anterior and lateral compartments. Emergent single-incision fasciotomy was performed four hours after diagnosis. The patient was treated successfully without any neuromuscular dysfunction. An early and accurate diagnosis is important to avoid the delay of treatment and development of neuromuscular dysfunction.

## 1. Introduction

Well leg compartment syndrome (WLCS) is a rare but severe complication after the surgery in lithotomy position. The overall incidence is estimated at 1 in 3500 cases; however, only less than 25 bilateral WLCS cases have been previously reported [1–4]. A prompt diagnosis and surgical intervention is necessary because the delay of treatment could cause irreversible muscle necrosis which results in limb dysfunction or amputation. Although two-incision technique to release all four compartments is recommended, fasciotomy itself is associated with a high incidence of acute and long-term complications [5, 6].

Here, we report the case of bilateral WLCS following the surgery in lithotomy position. Only anterior and lateral compartments were affected and successfully treated with single-incision fasciotomy.

## 2. Case Presentation

The patient was a 50-year-old male, 173 cm tall, and 85 kg in weight (body mass index (BMI): 27.7 kg/m^2^). He had a medical history of urinary tract cancer, type 2 diabetes mellitus, hypertension, and Hashimoto's disease. He underwent a robot-assisted radical cystectomy in lithotomy position. The total operation time was 419 min. The operative position was lithotomy position with his lower leg flexed and elevated by soft stirrups. Continuous compression devices on both calves were used for venous thrombosis prophylaxis throughout the procedure. No bleeding-promoting drug was used before and after the surgery.

**Figure 1:**
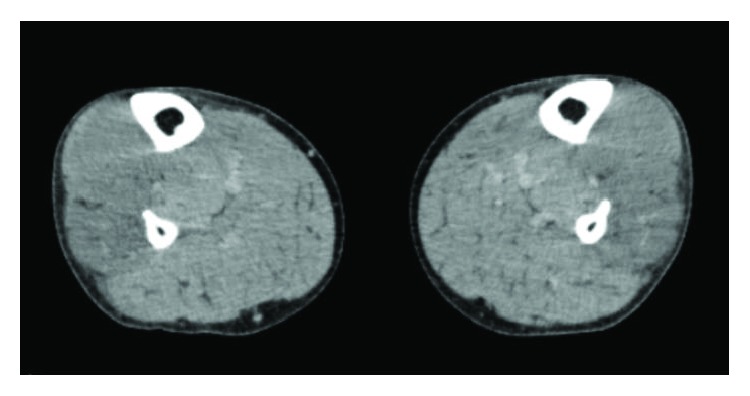
Contrasting computed tomography of the lower legs showed swelling and decreased enhancement of the muscles in the anterior and lateral compartments.

Sixteen hours after the surgery, he complained of severe bilateral lower leg pain and swelling. Initial evaluation of lower extremities revealed foot drop, swelling and tightness of the anterolateral aspects, and stretch pain on passive ankle planter flexion. No remarkable finding was appreciated on the posterior aspects of his lower legs. Serum creatine kinase was elevated to at 28000 U/l. The compartment pressure was measured by an arterial line set with simple 18-gauge needle under the diastolic blood pressure of 98 mmHg. The measurement was performed at three places of each compartment, and the average value was recorded. The anterior and lateral compartment pressures in both legs had increased to 200 mmHg despite normal posterior compartment pressure (35 mmHg) or thigh compartment pressure (35 mmHg). Contrasting computed tomography (CT) showed swelling of the bilateral muscles in the anterior and lateral compartments without contrasting effect compared to the posterior compartments (Figure 1). Based on these findings, WLCS localized in the anterior and lateral compartments was diagnosed.

**Figure 2:**
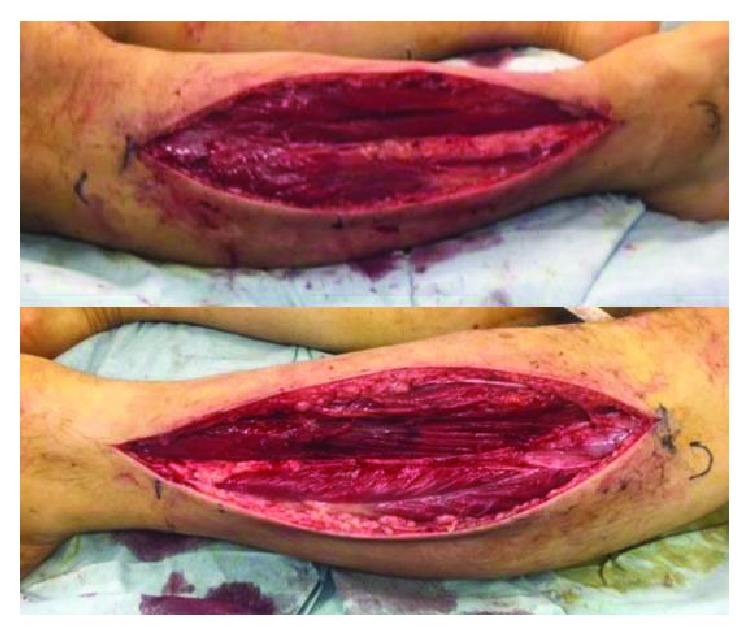
Single-incision fasciotomy was adapted to release the anterior and lateral compartments.

**Figure 3:**
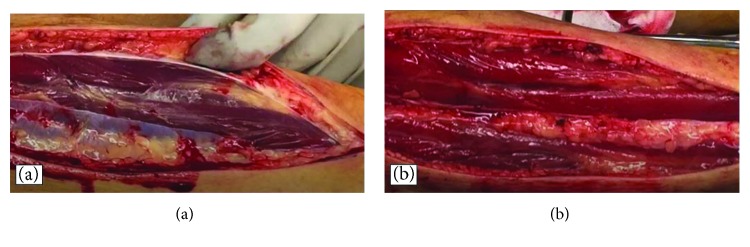
(a) Discolored muscles in the anterior compartment immediately after fasciotomy. (b) Improvement of muscle discoloration five minutes after the release of the anterior compartment.

**Figure 4:**
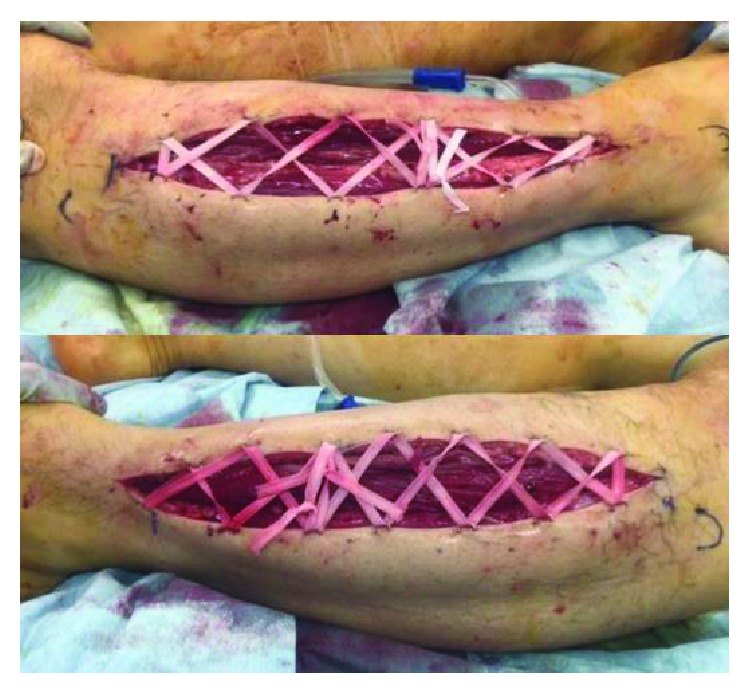
Shoe-race sutures were used to close the wound gradually.

An emergency fasciotomy was performed twenty hours after initial surgery. Anterior and lateral compartments were released with single incision (Figure 2). Discoloration of the muscles improved within a few minutes after the fasciotomy (Figure 3). Shoe-race procedure was added to prepare for secondary wound closure (Figure 4). Symptoms such as unbearable pain or decreased sensation were drastically improved after the fasciotomy. The serum creatine kinase decreased and normalized eight days after the surgery. He recovered well without any motor and sensory dysfunction in both lower extremities. The fasciotomy wound was closed on the ninth postoperative day without additional stage procedure. Three months after the surgery, he had no neuromuscular dysfunction.

## 3. Discussion

WLCS was first reported in 1979 as a severe complication after the surgery in lithotomy position [7]. It is rare, with almost one in every 3500 cases, but the delay in diagnosis and treatment may require lower leg amputation or result in death [1, 4]. The perfusion of the lower legs decreases in lithotomy position, which could cause muscle necrosis and massive edema due to ischemia [8]. Thus, the pressure within the compartment increases and affects the blood supply, leading to the vicious cycle.

Diagnosis of compartment syndrome is usually described as a clinical diagnosis that includes signs and symptoms of paresthesia, pain, pain on passive stretch, and tightness. Pulselessness and pallor are observed in advanced stage. The serum creatine kinase measurement and imaging study including contrasting CT or MRI may also aid the diagnosis. Although MRI can detect a small change of muscle, it is not suitable for diagnosis of emergent case due to the time commitment [9]. If the acute compartment syndrome is clinically suspected, the compartment pressure should be measured.

Compartment pressure is measured with several devices [9, 10]. Hammerberg et al. reported that slit catheter, side-ported needle, 18-gauge needle may be used with confidence [11]. The recent accepted value for diagnosis of compartment syndrome is 30 mmHg within the diastolic blood pressure [12–14]. Absolute value of 45 mmHg is also proposed [15]. Whitney et al. reported that reliance on one-time intracompartmental pressure measurements can overestimate the rate of compartment syndrome [16]. Repetitive measurement on several compartments is required for accurate diagnosis. In our case, the use of thin needle may produce erroneously the high value. However, additional measurement was determined to be unnecessary because early diagnosis without relying too much on compartment pressure measurement is important to avoid the delay of treatment. WLCS localized in the anterior and lateral compartments was the most probable diagnosis considering the relatively high compartment pressure.

The anterior compartment is the most commonly involved in acute compartment syndrome because it is rigidly surrounded by the tibia, fibula, interosseous septum, and fascia [17, 18]. The lateral compartment is the second most commonly involved. In our case, the patient's complaint, physical examination, and imaging study strongly suggested that only the anterior and lateral compartments were affected. Compartment pressure measurement also supported this idea. Therefore, we diagnosed this case with WLCS localized in the anterior and lateral compartments.

Definitive treatment for WLCS is emergent fasciotomy to decompress the compartments. As little as 8 hours of critical ischemia results in irreversible damage to the muscles and nerves [19]. Both single- and double-incision fasciotomy techniques have been recommended for releasing all 4 compartments of the lower extremity [18]. Single-incision technique is usually selected for fracture cases demanding internal fixation through another incision. 20 to 25 cm longitudinal incision is necessary for sufficient decompression [18]. However, fasciotomy itself is associated with a high incidence of acute and long-term complications such as nerve damage, bleeding, wound infection, altered sensation, continuing pain, eczematous changes, pruritus, discoloration, and recurrent ulceration [5, 6]. We chose the single-incision fasciotomy for anterior and lateral compartment release based on the localization of the affected compartments.

**Figure 5:**
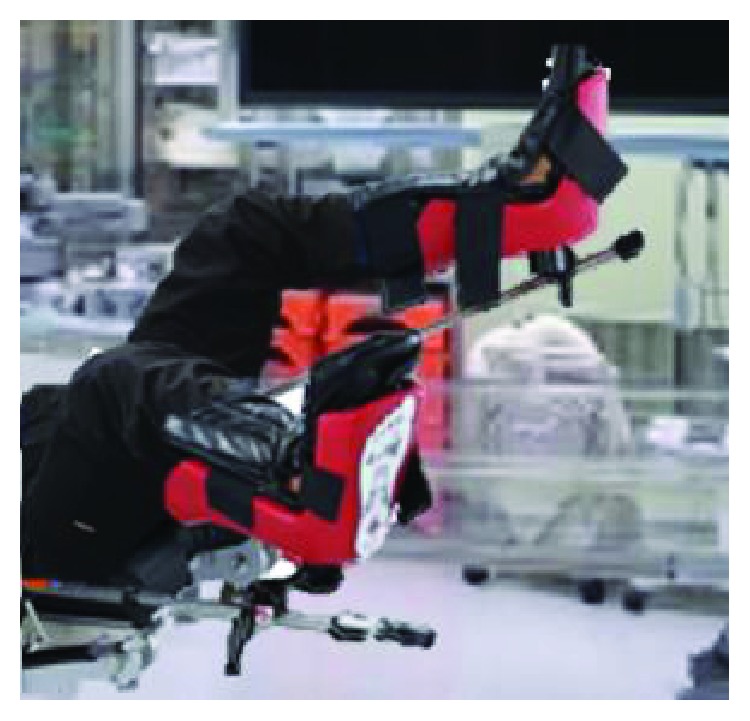
The recommended lithotomy position with not too much bending of the knee and hip joint. The same soft stirrups as this patient are used.

The time to fasciotomy has a great impact on the outcome of WLCS. For the early recognition of WLCS, the clinician's awareness of the risk factors is important. In lithotomy position, the mean arterial pressure decreases at the toe by 0.78 mmHg/cm as a result of leg elevation [8]. Furthermore, the lower extremity systolic blood pressure drops by the degree of hip flexion and leg height, which can lead to ischemia [4]. Flexed hip and knee joint may cause the kinking of veins and increase the venous pressure [20]. The direct compression of the lower limbs from solid device also cause the increase of compartment pressure. According to these previous studies, we suggest that the desirable lithotomy position should fulfill the condition of hip or knee flexion not beyond 90 degrees, hip abduction less than 45 degrees, and neutral hip rotation (Figure 5). In addition, releasing the leg from support every 2 hours for a short time is recommended to relieve the direct compression of the calves when operating time is estimated to be more than 4 hours [3]. Other risk factors for developing WLCS include the use of intermittent pneumatic compression devices, ankle dorsiflexion position, muscular lower limbs, solid leg holders, intraoperative hypotension, hypovolemia, and peripheral vascular disease. For patients suspected of WLCS with these risk factors, prompt diagnosis and surgical management are imperative.
